# Hypoxia disrupts proteostasis in *Caenorhabditis elegans*

**DOI:** 10.1111/acel.12301

**Published:** 2014-12-16

**Authors:** Emily M Fawcett, Jill M Hoyt, Jenna K Johnson, Dana L Miller

**Affiliations:** 1 Graduate Program in Molecular and Cellular Biology, University of Washington School of MedicineSeattle, WA, 98195-7350, USA; 2 Department of Biochemistry, University of Washington School of MedicineSeattle, WA, 98195-7350, USA; 3 Department of Biology, Luther CollegeDecorah, IA, 52101, USA

**Keywords:** *C. elegans*, hypoxia, H_2_S, oxygen, Polyglutamine, protein aggregation, proteostasis

## Abstract

Oxygen is fundamentally important for cell metabolism, and as a consequence, O_2_ deprivation (hypoxia) can impair many essential physiological processes. Here, we show that an active response to hypoxia disrupts cellular proteostasis – the coordination of protein synthesis, quality control, and degradation that maintains the functionality of the proteome. We have discovered that specific hypoxic conditions enhance the aggregation and toxicity of aggregation-prone proteins that are associated with neurodegenerative diseases. Our data indicate this is an active response to hypoxia, rather than a passive consequence of energy limitation. This response to hypoxia is partially antagonized by the conserved hypoxia-inducible transcription factor, *hif-1*. We further demonstrate that exposure to hydrogen sulfide (H_2_S) protects animals from hypoxia-induced disruption of proteostasis. H_2_S has been shown to protect against hypoxic damage in mammals and extends lifespan in nematodes. Remarkably, our data also show that H_2_S can reverse detrimental effects of hypoxia on proteostasis. Our data indicate that the protective effects of H_2_S in hypoxia are mechanistically distinct from the effect of H_2_S to increase lifespan and thermotolerance, suggesting that control of proteostasis and aging can be dissociated. Together, our studies reveal a novel effect of the hypoxia response in animals and provide a foundation to understand how the integrated proteostasis network is integrated with this stress response pathway.

## Introduction

Fluctuations in O_2_ availability are common in nature. Effective O_2_ concentration declines with altitude, and steep concentration gradients of O_2_ occur in marine environments and wet soil because O_2_ is poorly soluble in water and diffuses slowly in aqueous solution. Animals have evolved a variety of physiological and behavioral responses to low O_2_ (hypoxia). Nevertheless, hypoxia can be quite damaging, as O_2_ availability contributes to cellular damage and death in human pathological conditions where blood flow is interrupted such as severe blood loss, stroke, and cardiovascular disease. Cellular damage from hypoxia can be mitigated by a preconditioning exposure, in which a nonlethal hypoxic event precedes the damaging insult (reviewed in Semenza, 2011). This suggests that there are endogenous cellular mechanisms that can protect against damaging effects of hypoxia when appropriately activated.

It has been suggested that cellular damage occurs when arterial blood O_2_ concentration drops below 5000 ppm O_2_ (Carreau *et al*., 2011). However, the O_2_ available to different tissues is not uniform, and the sensitivity of different cell types to withstand hypoxia can vary dramatically. Tumor cells are particularly resistant to hypoxia, likely as an adaptation to poor O_2_ delivery in tumors. In fact, tumor hypoxia is strongly associated with poor prognosis and resistance to therapy (reviewed in Brown, 2007). There is great need to understand the diversity and integration of cellular responses to hypoxia. It is technically quite difficult to precisely measure or experimentally control cellular O_2_ concentrations in living mammals. We therefore have used *Caenorhabditis elegans* to investigate responses to specific hypoxic conditions. In this animal, all cells are directly exposed to the gaseous environment (Shen & Powell-Coffman, 2003). This allows for precise control of cellular O_2_ availability in a genetically tractable model, without the confounding effects of compensatory responses that increase blood flow to hypoxic tissues, which are common in larger animals.

The physiological response to hypoxia depends greatly on the amount of O_2_ that is available, as has been well demonstrated in the nematode *C. elegans*. *C. elegans* is broadly tolerant to hypoxia and can continue development and reproduction in as little as 5000 ppm O_2_ (Nystul *et al*., 2003; Miller & Roth, 2009). In anoxia (operationally defined here as < 10 ppm O_2_), *C. elegans* enters into a reversible state of suspended animation, in which all observable biological processes arrest (Padilla *et al*., 2002; Nystul *et al*., 2003). Upon return to normoxia (which we define as room air, 210 000 ppm O_2_), animals reanimate and resume normal biological activity without apparent consequence.

Curiously, there are a range of O_2_ conditions in which *C. elegans* can neither induce suspended animation nor continue development. In 1000 ppm O_2_, isolated embryos continue to develop and die with gross morphological and developmental defects (Nystul *et al*., 2003). Exposure to 1000 ppm O_2_ is not lethal after embryogenesis, but instead induces diapause in which development and reproduction – but not movement and other biological activities – reversibly arrest (Miller & Roth, 2009). These observations support the idea that there are multiple distinct but highly coordinated responses to hypoxia.

Hypoxia extends lifespan in both *C. elegans* and *Drosophila* (Rascon & Harrison, 2010; Leiser *et al*., 2013). These observations suggest that hypoxia responses integrate mechanistically with longevity-associated cellular processes. Many studies suggest that proteostasis is essential to prevent cellular decline associated with aging (reviewed in Morley *et al*., 2002; Taylor & Dillin, 2011). Proteostasis is the coordination of protein translation, folding, quality control, trafficking, and degradation that is essential to maintain the proteome in a functional state. Hypoxia can impact many, if not all, of the cellular processes involved in proteostasis. In flies, turtles, and mammalian cell culture, protein translation arrests in hypoxia (Teodoro & O'Farrell, 2003; Liu & Simon, 2004; Liu *et al*., 2006). Chaperones, heat shock proteins, and the unfolded protein response are activated by hypoxia in mammalian cells as well as *C. elegans* (Koumenis *et al*., 2002; Wouters & Koritzinsky, 2008; Mao & Crowder, 2010; Powell-Coffman, 2010). Decreasing translation and upregulating heat shock proteins might be predicted to maintain proteostasis. However, O_2_ is required for correct disulfide bond formation in protein folding and in *Drosophila*, protein turnover arrests upon O_2_ deprivation (Teodoro & O'Farrell, 2003), which could impair cellular proteostasis. Thus, the effects on global proteostasis are not easily predicted.

The gas hydrogen sulfide (H_2_S) has been shown to improve outcome in several models of hypoxic and ischemic damage in mammals (reviewed in Nicholson & Calvert, 2010). Mice exposed to H_2_S survive otherwise lethal hypoxia (Blackstone & Roth, 2007; Elrod *et al*., 2007). In preclinical mammalian models, treatment with H_2_S improves outcome in myocardial infarct and cerebral ischemic injury (Predmore & Lefer, 2011; Liu *et al*., 2012). One possibility is that H_2_S signaling impinges on pathways similar to those that mediate the protective effects of hypoxic preconditioning. Consistent with this view, H_2_S stabilizes and activates HIF-1 in both mice and *C. elegans* (Budde & Roth, 2010), and HIF is important for hypoxic preconditioning in myocardial infarct (Liu *et al*., 2010; Sarkar *et al*., 2012). Curiously, in *C. elegans*, different genes are regulated by *hif-1* in H_2_S and hypoxia (Miller *et al*., 2011), suggesting that the protective effects of H_2_S are not simply a result of activating the HIF-mediated response to hypoxia.

In this study, we measured the functional effect of hypoxia on proteostasis in living animals using the nematode *C. elegans*. Our results indicate that in specific hypoxic conditions, there is an active cellular response that perturbs proteostasis. The perturbation of proteostasis persists even when O_2_ is restored. The *hif-1* transcription factor is not required for this aspect of the hypoxia response. Instead, we show *hif-1* partially suppresses the effect of hypoxia on proteostasis in some conditions. We also demonstrate that treatment with H_2_S can both prevent and reverse detrimental effects of hypoxia on proteostasis. Our observation that H_2_S protects against hypoxia is reminiscent of the situation in mammals, suggesting that the functional integration of hypoxia and H_2_S responses is conserved.

## Results

**Figure 1:**
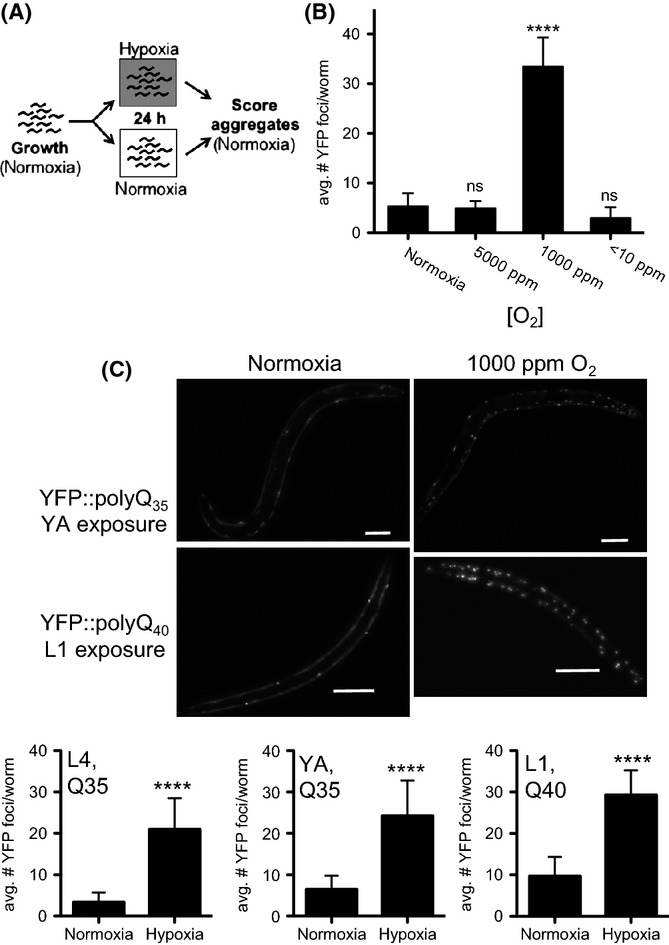
Hypoxia induces polyglutamine protein aggregation. (A) Experimental design. Synchronized populations of YFP::polyQ_35_ animals were grown in normoxia, then the population was divided and half were exposed to hypoxia. The number of YFP foci was scored immediately upon removal from hypoxia. (B) Specific hypoxic O_2_ concentrations induce polyglutamine protein aggregation. Cohorts of young adult YFP::polyQ_35_ were exposed to atmosphere containing the indicated concentration of O_2_. YFP foci were counted immediately upon removal. (C) Polyglutamine protein aggregation is induced by exposure to 1000 ppm O_2_. Fluorescence microscopy images show YFP::polyQ_35_ (top) and YFP::polyQ_40_ (bottom) in live *Caenorhabditis elegans* after 24 h in either normoxia (left) or 1000 ppm O_2_ (right). Bar graphs (below*)* show the mean number of YFP::polyQ foci after 24 h in hypoxia (1000 ppm O_2_) or normoxia (RA = 210 000 ppm O_2_). Animals were exposed as either as fourth-stage larvae (L4, left), young adult (YA, middle), or first-stage larvae (L1, right). Q35 = YFP::polyQ_35_; Q40 = YFP::polyQ_40_. In all panels, graphs show mean ± SD. Each cohort included at least 30 animals. Statistical comparisons were between groups exposed to hypoxia and normoxic controls: ****P-value < 0.0001; ns, not significant. Summary of data from replicate experiments is included in Table S1 (Supporting information).

In *C. elegans*, cells acquire O_2_ directly from the environment, rather than by active transport through a vascular system. Therefore, in contrast to larger animals, *C. elegans* do not respond to hypoxia with adaptations that improve delivery of O_2_ to cells, such as increased respiration or heart rate. We took advantage of this feature of *C. elegans* biology and exposed animals to constructed environments with defined concentrations of O_2_ to precisely control cellular O_2_ (Nystul & Roth, 2004; Fawcett *et al*., 2012). To evaluate the effects of hypoxia on proteostasis *in vivo*, we utilized a well-established polyglutamine protein model. In these animals, the yellow fluorescent protein (YFP) is fused to a series of glutamine residues and expressed in the body wall muscle. We refer to this transgene as YFP::polyQ_x_ (the subscript indicates the number of glutamine residues fused to YFP). YFP::polyQ_x_ is soluble and diffuse throughout the muscle cells when first expressed, but aggregates as proteostasis mechanisms fail, forming bright fluorescent foci (Fig.1C, for example). Thus, the localization of YFP::polyQ_x_ is a read-out of proteostasis efficiency *in vivo*. This model has been validated in studies that have determined the effects of aging, genetic disruption of quality control machinery, and osmotic stress on the proteostasis network (Morley *et al*., 2002; Gidalevitz *et al*., 2006; Moronetti Mazzeo *et al*., 2012).

### Specific hypoxic condition induces aggregation of polyglutamine proteins

We exposed animals expressing YFP::polyQ_35_ to hypoxia as first-day adults, before the onset of age-associated protein aggregation, to determine the effect of hypoxia on proteostasis (schematized in Fig.1A). *C. elegans* survive exposure to all O_2_ conditions tested in our experiments. We observed that animals exposed to environments with as little as 5000 ppm O_2_ (a 40-fold reduction in O_2_ from room air, 210 000 ppm O_2_) showed no difference in the number of YFP foci compared to controls that remained in room air (Fig.1B). This suggests that proteostasis is effectively maintained in this condition, even though the decrease in O_2_ causes a severe decrease in metabolic and developmental rate (Miller & Roth, 2009; Van Voorhies, 2009).

In contrast to the situation in 5000 ppm O_2_, the number of YFP foci increased dramatically in animals exposed to 1000 ppm O_2_ (Fig.1B), suggesting that proteostasis has been disrupted. YFP foci did not form in 1000 ppm O_2_ in YFP::polyQ_0_ control animals that express YFP without a polyglutamine tract indicating that the effect of hypoxia depends on the polyglutamine tract. Animals also developed increased YFP::polyQ_35_ foci when they were exposed to 1000 ppm O_2_ as fourth-stage larvae (L4) (Fig.1C), indicating that this response was robust across developmental stages. Animals exposed to 1000 ppm O_2_ enter into a developmental and reproductive diapause (Miller & Roth, 2009) and are therefore developmentally younger than controls that remain in room air.

We did not observe increased formation of YFP foci in animals exposed to anoxia (Fig.1B). These results suggest that the disruption of proteostasis in 1000 ppm O_2_ is an active consequence of the response to hypoxia, and not simply a passive consequence of decreased aerobic energy production. In anoxia, *C. elegans* enter into a state of suspended animation, which is poorly understood mechanistically. We cannot exclude the possibility that suspended animation itself protects the proteostasis network. Another possible interpretation of this result is that the process of aggregation itself is an active process, requiring a functioning cellular metabolic state. These specific effects on proteostasis in distinct O_2_ concentrations are yet another piece of evidence that there are distinct physiological responses to different hypoxic conditions.

Aggregation of YFP::polyQ_x_ occurs in a polyglutamine tract length- and age-dependent manner in normoxic conditions (Morley *et al*., 2002). We therefore considered the possibility that hypoxia could have an age-dependent effect on proteostasis. To test this possibility, we compared the effects of hypoxia on strains expressing either YFP::polyQ_35_ or YFP::polyQ_40_. In normoxia, YFP::polyQ_35_ does not begin to aggregate until the animals are adults, whereas YFP::polyQ_40_ protein is more aggregation-prone and forms foci starting at L1/L2. We found that exposure to 1000 ppm O_2_ did not induce aggregation of YFP::polyQ_35_ in first-stage larvae (L1), in contrast to our previous results showing increased aggregation in both L4 and adults (Fig.1C and data not shown). However, we did observe an increase in the number of fluorescent foci when YFP::polyQ_40_ animals were exposed as L1 (Fig.1C). We conclude that the effect of hypoxia on proteostasis is similar at all developmental stages. The difference between the effect of hypoxia on L1 animals expressing YFP::polyQ_35_ and YFP::polyQ_40_ suggests that these proteins are differentially vulnerable to how the proteostasis network is perturbed in hypoxia. For example, one possibility is that hypoxia disrupts proteostasis in a manner that promotes the growth of aggregates but not the formation of new aggregate seeds. The fact that protein aggregation is induced by hypoxia throughout life indicates that hypoxia has age-independent effects on proteostasis.

### Hypoxia enhances proteotoxicity of neurodegeneration disease models in *C. elegans*

**Figure 2:**
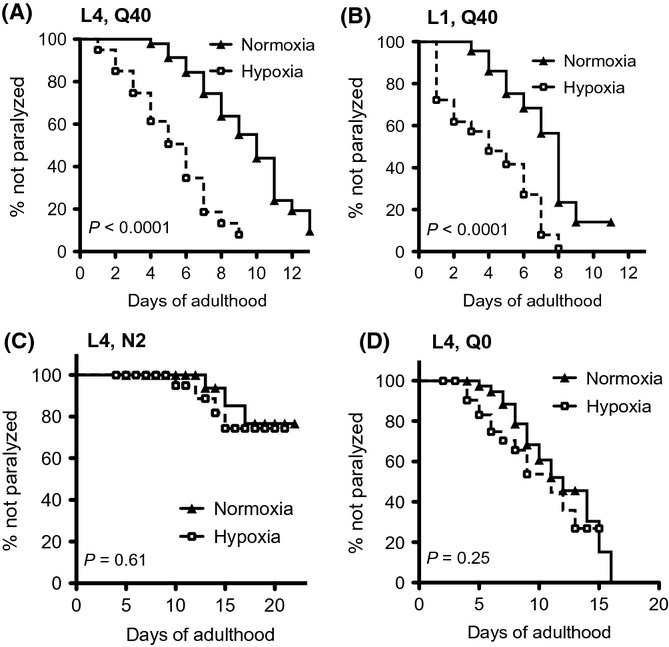
Hypoxia accelerates paralysis associated with expression of polyglutamine proteins. YFP::polyQ_x_ animals exposed to hypoxia become paralyzed sooner than normoxic controls. Age-matched animals were exposed to 1000 ppm O_2_ for 24 h and then returned to normoxia. Paralysis was scored daily. (A) Paralysis of YFP::polyQ_40_ animals exposed as L4. (B) Paralysis of YFP::polyQ_40_ animals exposed as L1. (C) Paralysis of N2 (wild-type) animals exposed as L4. (D) Paralysis of YFP::polyQ_0_ animals exposed as L4. Each cohort included 30–50 animals. *P*-values on each graph compare hypoxia to normoxia using Kaplan–Meyer statistics. Summary of data from replicate experiments is included in Table S2 (Supporting information).

It has been proposed that aggregation of proteins involved in neurodegeneration is a cytoprotective response to sequester more toxic, smaller aggregates. One possibility is that protein aggregation in hypoxia might similarly be a protective mechanism to reduce toxic effects of unfolded or damaged proteins. We evaluated this hypothesis by measuring the proteotoxicity of YFP::polyQ_x_ after hypoxia. In room air, YFP::polyQ_x_ toxicity leads to age-associated disruption of muscle cell function and paralysis. We reasoned that if increased protein aggregation is a cytoprotective response to hypoxia, then animals exposed to hypoxia would maintain muscle function as long as, or longer than, controls. To assess this, we measured the onset of paralysis in animals exposed to 1000 ppm O_2_. We observed that both YFP::polyQ_40_ and YFP::polyQ_35_ animals become paralyzed sooner when exposed to 1000 ppm O_2_, but not 5000 ppm O_2_ (Fig.2A,B, Fig. S1, and data not shown). Importantly, hypoxia does not induce paralysis in wild-type (N2) or YFP::polyQ_0_ animals, suggesting that the proteotoxicity we observe in YFP::polyQ_x_ animals is due to cytotoxicity associated with polyQ_x_ (Fig.2C,D). We conclude that proteotoxicity of polyglutamine proteins is enhanced by hypoxia.

**Figure 3:**
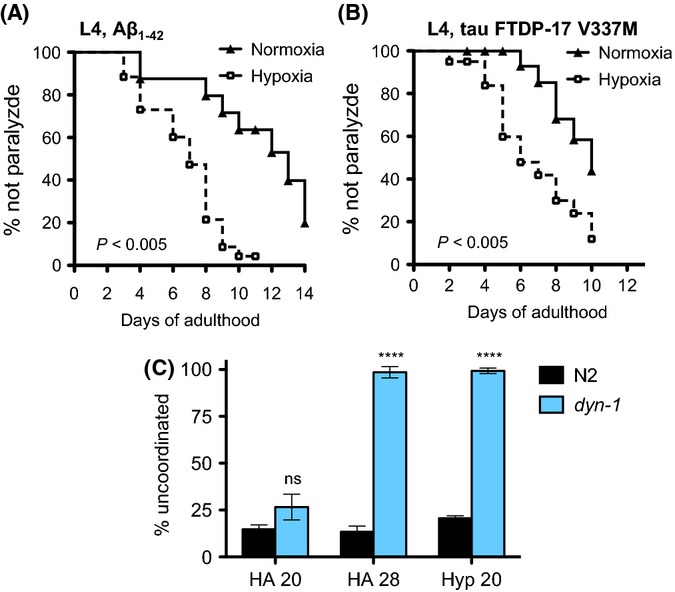
Hypoxia accelerates paralysis associated with aggregation-prone and metastable proteins. (A) Wild-type animals expressing human Aβ_1–42_ in body wall muscle become paralyzed more rapidly when exposed to 1000 ppm O_2_ as L4. (B) *Caenorhabditis elegans* expressing the human V337M tau variant associated with FTDP-17 in neurons become paralyzed more rapidly when exposed to 1000 ppm O_2_ as L4. (C) Metastable proteins are less functional after animals are exposed to 1000 ppm O_2_. Percentage of uncoordinated *dyn-1(ky51)* and WT animals at the permissive (20°C) or nonpermissive (28°C) temperature exposed as L4s to hypoxia (1000 ppm O_2_) for 24 h or maintained in normoxia. ****P < 0.0001 when compared to wild-type; ns, not significant. Each cohort included 30–50 animals. Summary of data from replicate experiments is included in Table S3 (Supporting information).

To further establish that hypoxia causes a general defect in proteostasis, we examined whether other aggregation-prone proteins were also affected by exposure to hypoxia. We first measured the effect of hypoxia on animals expressing Aβ_1–42_ in body wall muscle (Link, 1995). In *C. elegans,* Aβ_1–42_ forms aggregates similar to amyloid plaques associated with Alzheimer's disease in humans, and leads to age-associated muscle dysfunction and paralysis. We found that, similar to the YFP::polyQ_x_ model, animals expressing Aβ_1–42_ became paralyzed sooner when exposed to 1000 ppm O_2_ (Fig.3A). Based on this finding, we conclude that hypoxia-induced disruption in proteostasis is not specific to YFP::polyQ_x_.

Both the YFP::polyQ_x_ and Aβ_1–42_ transgenes are expressed in the body wall muscle. This raises the possibility that hypoxia-induced disruption of proteostasis is specific to this tissue. To address this possibility, we measured the effect of hypoxia in neurons of animals that express the human tau(V337M) protein variant from the *aex-3* pan-neuronal promoter. This mutation causes a progressive neurodegenerative disease in humans (frontotemporal dementia with parkinsonism linked to chromosome 17). The tau(V337M) variant reduces binding affinity of tau to microtubules, accelerates tau aggregation, and leads to age-associated paralysis (Kraemer *et al*., 2003). We found that animals expressing tau(V337M) in the nervous system became paralyzed more rapidly after exposure to 1000 ppm O_2_ (Fig.3B). We conclude that hypoxia disrupts global proteostasis in both neurons and muscle, supporting the idea that exposure to hypoxia results in an organism-wide disruption of proteostasis.

Thus far, the models we have investigated rely on transgenic expression of exogenous aggregate-prone proteins. To further test our model that hypoxia responses lead to a general disruption of proteostasis, we utilized animals with temperature sensitive (ts) mutations in the neuronal dynamin protein DYN-1. The *dyn-1(ky51)* ts mutant allele encodes a metastable DYN-1 protein. *dyn-1(ts)* mutant animals are uncoordinated at the restrictive temperature (28°C), but have normal motility at the permissive temperature (20°C). Conditions that disrupt proteostasis prevent the proper folding of the metastable DYN-1, causing *dyn-1(ts)* animals to become paralyzed even at the permissive temperature (Clark *et al*., 1997; Gidalevitz *et al*., 2006). We predicted that *dyn-1(ts)* animals exposed to 1000 ppm O_2_ would become paralyzed at the permissive temperature as a result of the hypoxia-induced disruption of proteostasis.

We monitored the motility of *dyn-1(ts)* mutant animals at 20°C to assess the effect of hypoxia on proteostasis. Consistent with our hypothesis that hypoxia disrupts proteostasis, we found that animals exposed to hypoxia for 24 h at the permissive temperature displayed a severe impairment of motility (Fig.3C). The same hypoxic conditions had no effect on wild-type (N2) animals. We conclude that the response to hypoxia disrupts the cellular folding environment and impairs the ability of the DYN-1 protein to function. Taken together with our experiments using YFP::polyQ_x_, Aβ_1–42_, and Tau, our results support a model in which exposure to hypoxia results in a widespread loss of proteostasis.

### *hif-1* is necessary but not sufficient to protect against hypoxia-induced protein aggregation

In studies of age-induced changes to proteostasis, changes in expression of proteasome subunits, autophagy, and chaperones are commonly noted (Lapierre *et al*., 2011; Vilchez *et al*., 2012; Taylor & Dillin, 2013). Insofar as we observed a functional effect on proteostasis, we hypothesized that exposure to hypoxia may also change the expression of key components of the proteostasis network. To address this possibility, we measured the abundance of transcripts of genes that are modified in other conditions that alter proteostasis using qRT–PCR. We did not observe any changes of transcript abundance after hypoxia for genes encoding several core proteasome subunits, or those genes critically involved in autophagy, TOR signaling, or the unfolded protein response (Fig. S2). This finding is in agreement with previously published microarray data of animals exposed to hypoxia, in which few changes to genes involved in proteostasis were observed (Shen *et al*., 2005).

**Figure 4:**
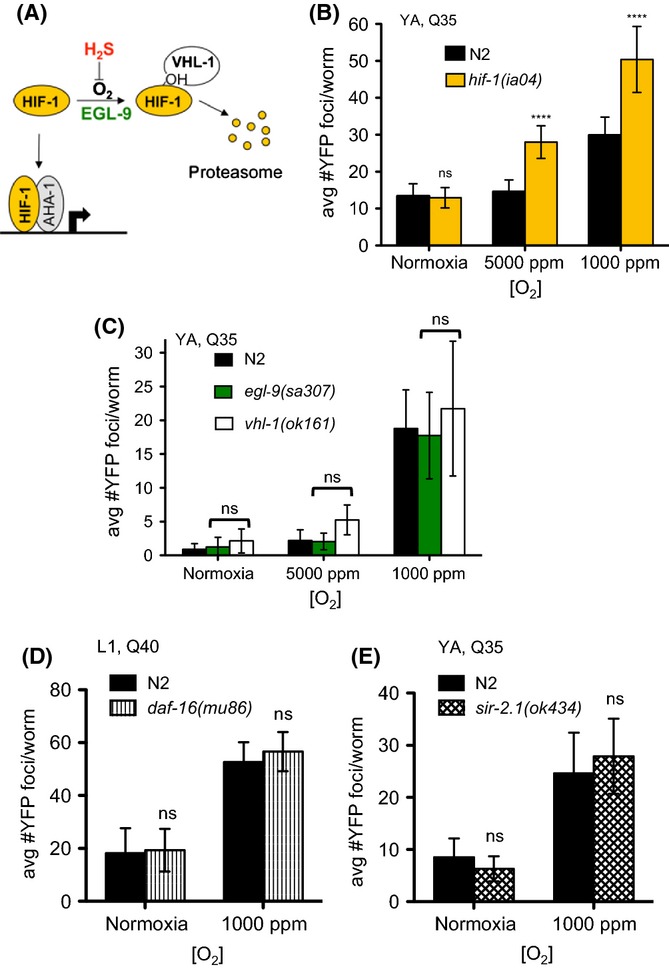
HIF-1 is necessary but not sufficient to protect against hypoxia-induced protein aggregation. (A) HIF-1 protein levels are regulated by O_2_ and H_2_S (reviewed in Semenza, 2011). In the presence of O_2_, the transcription factor HIF-1 is hydroxylated by the prolyl hydroxylase EGL-9. Hydroxylated HIF-1 is recognized by the E3 ubiquitin ligase von Hippel–Lindau protein 1 (VHL-1) and targeted for degradation by the proteasome. In hypoxia, the hydroxylation reaction is inefficient, resulting in accumulation of HIF-1 protein, which enters the nucleus and induces transcription. HIF-1 protein also accumulates in animals exposed to H_2_S, even when O_2_ is abundant (Budde & Roth, 2010). (B) HIF-1 is necessary to protect against protein aggregation in hypoxia. The number of YFP foci is greater in *hif-1(ia04); YFP::polyQ35* mutant animals than wild-type controls in both 5000 ppm O_2_ and 1000 ppm O_2_ but not normoxia. (C) HIF-1 is not sufficient to protect against hypoxia-induced protein aggregation. Mutations in negative regulators *egl-9* and *vhl-1* cause constitutive stabilization of HIF-1, even in normoxia. The number of YFP foci is not different from wild-type in *egl-9(sa307); YFP::polyQ*_*35*_ or *vhl-1(ok161);YFP::polyQ*_*35*_ mutant animals exposed to each hypoxic O_2_ concentration. (D) Hypoxia-induced protein aggregation is independent of the insulin-like/IGF signaling pathway. The number of YFP foci in *daf-16(mu86); YFP::polyQ*_*40*_ mutant animals is not different from wild-type after exposure to 1000 ppm O_2_. (E) Sirtuin activity does not regulate hypoxia-induced protein aggregation. The number of YFP foci in *sir-2.1(ok434); YFP::polyQ*_*35*_ mutant animals is not different from wild-type after exposure to 1000 ppm O_2_. In all panels, graphs show mean ± SD error, each cohort contained at least 30 animals. Statistical comparisons were to wild-type controls in the same condition: ****P < 0.0001; ns, not significant. Summary of data from replicate experiments is included in Tables S4 and S6 (Supporting information).

Although we did not observe a change in transcription of core proteostasis factors, we predicted that there would be genetic factors that mediate the effect of hypoxia on proteostasis if it was a result of the active response to hypoxia. We considered HIF-1 as a candidate for such a factor. HIF-1 is a well-studied and highly conserved bHLH transcription factor that coordinates the transcriptional response to hypoxia in metazoans, including *C. elegans* (reviewed in Semenza, 2009; Powell-Coffman, 2010). HIF-1 protein stability and transcriptional activity are directly regulated by O_2_ availability (schematized in Fig.4A). Earlier studies demonstrate that constitutive activation of HIF-1 reduces the toxicity of the aggregation-prone protein Aβ_1–42_ in normoxia (Mehta *et al*., 2009).

We crossed YFP::polyQ_x_ transgenes into *hif-1(ia04)* mutant animals to determine whether *hif-1* is required for the effect of hypoxia on proteostasis. The *ia04* allele is a deletion of exons 2-4 and predicted molecular null (Jiang *et al*., 2001). In 5000 ppm O_2_, *hif-1(ia04)* mutant embryos die, while larvae and adults precociously enter hypoxia-induced diapause (Nystul & Roth, 2004; Miller & Roth, 2009). We found the number of YFP::polyQ_35_ foci increased in *hif-1(ia04)* animals exposed to 5000 ppm O_2_ (Fig.4B), although there was no change in the number of foci when wild-type animals were exposed to the same conditions (see also Fig.1B). Importantly, we did not observe any difference in the extent of age-associated aggregation of YFP::polyQ_35_ in *hif–1*(*ia04*) mutant animals compared to wild-type controls (Fig.4B). These experiments indicate that HIF-1 activity is necessary to stop the perturbation of proteostasis in wild-type animals exposed to 5000 ppm O_2_.

Our observation that wild-type animals, with fully functional HIF-1, cannot maintain proteostasis in 1000 ppm O_2_ (Fig.1B) suggests that *hif-1* is not sufficient to prevent the disruption of proteostasis in these conditions, in contrast to the situation in 5000 ppm O_2_ (Fig.4B). This observation is consistent with earlier studies showing that HIF-1 mediates physiological responses to hypoxic O_2_ concentrations ≥ 5000 ppm O_2_, but not in more severe hypoxia. For example, *hif-1* is required for survival of embryos exposed directly to 5000 ppm O_2_ but has no effect on viability of embryos exposed to 1000 ppm O_2_ or anoxia (Nystul *et al*., 2003; Miller & Roth, 2009). Similarly, *hif-1* is required for continued postembryonic development and reproduction in 5000 ppm O_2_ (Miller & Roth, 2009).

The fact that *hif-1* is necessary to prevent hypoxia-induced protein aggregation in 5000 ppm O_2_ but not able to protect proteostasis in wild-type animals exposed to 1000 ppm O_2_ could indicate that the perturbation of proteostasis is mechanistically different in these two conditions. In this scenario, we expect that disrupting *hif-1* would not affect protein homeostasis in 1000 ppm O_2_. To test this, we counted the number of fluorescent foci in *hif–1(ia04)* mutant animals expressing YFP::polyQ_35_ after exposure to 1000 ppm O_2_. We observed more aggregates in *hif–1(ia04)* mutant animals exposed to 1000 ppm O_2_ than in wild-type controls (Fig.4B). These data suggest that activation of HIF-1 antagonizes the disruption of proteostasis, at least partially, in 1000 ppm O_2_ as well as preventing a perturbation in proteostasis in 5000 ppm O_2_.

Increased HIF-1 activity delays age-induced proteotoxicity of both YFP::polyQ_35_ and Aβ_1–42_ (Mehta *et al*., 2009). Our data show that HIF-1 activity can reduce protein aggregation after exposure to hypoxia. However, we observed no difference between the rate of hypoxia-induced paralysis in *hif-1*(*ia04*) mutant animals and wild-type controls that express YFP::polyQ_35_ (Fig. S3). One possibility is that mutations in *hif-1* separate the effects of proteostasis on protein aggregation and proteotoxicity in hypoxia. However, technical differences in the assays used to measure protein aggregation and toxicity complicate this interpretation. The number of fluorescent foci is scored immediately after the exposure to hypoxia, whereas YFP::polyQ_x_-associated paralysis must be measured days after the exposure to hypoxia. Another important feature of the assays is that paralysis is a binary measurement – animals are either paralyzed or they are not. In contrast, the number of fluorescent foci in YFP::polyQ_x_ is quantitative, so there are more than two possible outcomes. We cannot rule out the possibility that, unlike the aggregation assay, the paralysis assay is simply not sensitive enough to detect partial changes in proteostasis.

The fact that *hif-1* has only a partial effect to prevent protein aggregation in 1000 ppm O_2_ could indicate that there are additional protective mechanisms needed to protect proteostasis in this condition. Alternatively, it is possible that *hif-1* could induce the necessary factors but that in these severe conditions *hif-1* is simply overwhelmed. In this scenario, we would expect that increasing the activity of HIF-1 would reduce the number of protein aggregates after exposure to hypoxia.

Mutations in either *egl-9* or *vhl-1* lead to the constitutive stabilization and increased transcriptional activity of *hif-1*, even in normoxia (schematized in Fig.4) (Epstein *et al*., 2001; Shen *et al*., 2006; Budde & Roth, 2010). The level of HIF-1 stabilization in these mutants is sufficient to reduce the toxicity of Aβ_1–42_ proteins in normoxia (Mehta *et al*., 2009) and dramatically increases expression of common *hif-1* reporters (Shen *et al*., 2006). We found that both *vhl-1(ok161)* and *egl-9(sa304)* mutant animals expressing YFP::polyQ_35_ accumulate the same number of foci as wild-type controls when exposed to 1000 ppm O_2_ (Fig.4C). This result argues that factors other than HIF-1 are required to protect against effects of 1000 ppm O_2_ on proteostasis.

The conserved transcription factor DAF-16 and the SIRT1 homolog SIR-2.1 are attractive candidates for factors that could be working with HIF-1 in hypoxia. DAF-16 interacts with HIF-1 and has been shown to regulate proteostasis and lifespan (Murphy *et al*., 2003; Shen *et al*., 2005; Leiser *et al*., 2013). Similarly, SIR-2.1 has been shown to regulate lifespan and plays a role in coordinating the maintenance of proteostasis under stress conditions (Kaeberlein *et al*., 1999; Parker *et al*., 2005; Raynes *et al*., 2012). To determine whether DAF-16 or SIR-2.1 contributes to hypoxia-induced protein aggregation, we crossed the YFP::polyQ_x_ transgenes into *daf-16(mu86)* and *sir–2.1(ok434)* mutant animals. The *daf–16(mu86*) allele, an 11 kb genomic deletion that removes nearly all of the winged-helix domain, is a presumed molecular null. The *sir–2.1(ok434)* allele contains a 1 kb deletion and an insertion resulting in a frameshift. When exposed to hypoxia, we observed that both *daf–16(mu86);*YFP::polyQ_40_ and *sir-2.1(ok434);*YFP::polyQ_40_ mutant animals developed as many aggregates as wild-type controls (Fig.4D,E). We therefore conclude that the effect of hypoxia on proteostasis is independent of the insulin/IGF like signaling pathway and *sir-2.1*.

### The response to hypoxia has long-lasting effects on proteostasis

**Figure 5:**
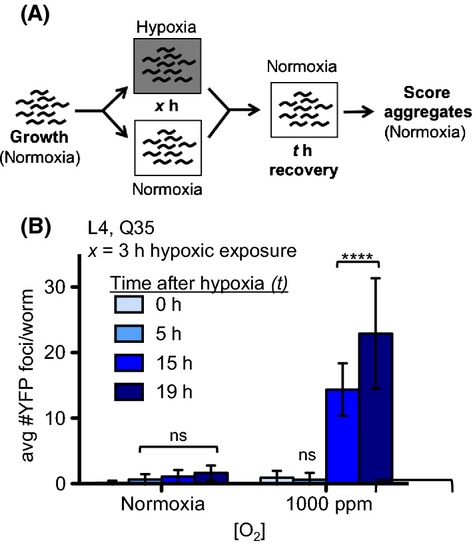
Exposure to hypoxia has long-lasting effects on proteostasis. (A) Experimental design. YFP::polyQ_35_ animals were grown in normoxia then transiently exposed to hypoxia for 3 h as L4. The number of YFP foci was scored after recovery in normoxia at each designated time (*t*) during recovery. (B) Short exposure to 1000 ppm O_2_ disrupts proteostasis after return to normoxia. No aggregates were observed immediately after YFP::polyQ_35_ animals were exposed to hypoxia, but the number of aggregates increased significantly more rapidly in the hypoxia-exposed cohort than controls during the recovery. Foci number was statistically compared to control animals at *t *=* *0 h: ****P < 0.0001; ns, not significant. Each cohort had at least 30 animals. Graphs show mean ± SD. Summary of data from replicate experiments is included in Table S5 (Supporting information).

We next investigated whether the perturbation of proteostasis in hypoxia was reversible, or if the damage had a lasting effect upon the return to normoxia. We reasoned that if proteostasis recovered after the hypoxic insult, the rate of protein aggregation would be the same in animals regardless of whether they had been exposed to hypoxia. One complicating factor is that the rate of aggregation depends partly on how many aggregates had already formed. To separate the effects of hypoxia from the effect of increased aggregate number, we exposed animals to 1000 ppm O_2_ for only 3 h (schematized in Fig.5A). There is no increase in the number of YFP::polyQ_35_ foci immediately after this short exposure to hypoxia. However, we found that the appearance of aggregates was accelerated in animals exposed to hypoxia (Fig.5B). The number of aggregates in control animals that remain in room air did not increase, confirming that there were no age-associated defects in proteostasis over the course of this experiment.

Our result suggests that response(s) to hypoxia (or the transition between hypoxia and normoxia) induces long-lasting defects in proteostasis that cannot be corrected in room air. Consistent with this hypothesis, animals expressing YFP::polyQ_40_ became paralyzed more rapidly after return to room air whether exposure was during L1 or L4 (Fig.2A,B). In both situations, increased protein aggregation was observed at the time of the hypoxic exposure, but animals became paralyzed at adulthood. Thus, although the duration of hypoxic insults and transitions between hypoxia and normoxia were the same for the L1 and L4 cohorts, the time between protein aggregation and toxicity was longer in the L1 cohort than for those animals exposed as L4. This result supports the idea that aggregation that occurs during exposure to hypoxia does not alone explain the tissue damage that leads to eventual paralysis.

### Adaptation to H_2_S protects against hypoxia-induced disruption of proteostasis

Many studies suggest an intimate relationship between proteostasis and aging. Accumulating evidence shows that H_2_S can effectively reduce cellular damage and death resulting from ischemia/reperfusion (I/R) injury in mammals (reviewed in Nicholson & Calvert, 2010). Moreover, H_2_S increases lifespan and thermotolerance in *C. elegans* (Miller & Roth, 2007).

**Figure 6:**
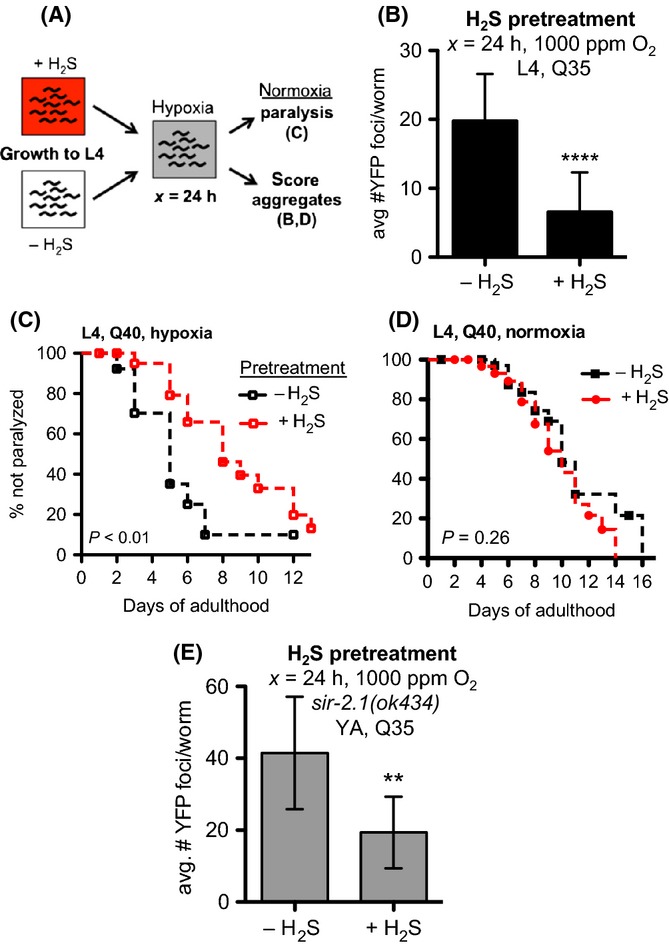
Adaptation to H_2_S protects against hypoxia-induced effects on proteostasis. (A) YFP::polyQ_x_ animals were grown in normoxia in the presence or absence of 50 ppm H_2_S and then transiently exposed to 1000 ppm O_2_ for 24 h. (B) Animals grown in H_2_S develop fewer aggregates in 1000 ppm O_2_. Animals were grown to L4 in H_2_S then exposed to hypoxia. Aggregates were counted immediately after exposure to hypoxia. (C) H_2_S pretreatment delays polyglutamine-associated paralysis after exposure to 1000 ppm O_2_. Animals were grown to L4 in H_2_S and then exposed to hypoxia. After return to normoxia (room air), paralysis was scored daily. (D) H_2_S does not alter age-associated paralysis induced by YFP::polyQ_40_. Animals were exposed to H_2_S for first 48 h of adulthood, and then paralysis was monitored in room air. (E) The effect of H_2_S on proteostasis in hypoxia is independent of *sir-2.1*. The number of YFP foci in *sir-2.1(ok434);YFP::polyQ*_*35*_ mutant animals after exposure to 1000 ppm O_2_ was decreased by pretreatment similar to wild-type. For all panels, graph shows mean number of foci with SD error bars, each cohort consisting of 30–40 animals. Statistical comparisons were to matched normoxic controls: ****P < 0.0001; **P < 0.005; ns, not significant. Summary statistics from replicate experiments are provided in Table S6 (Supporting information).

We considered the hypothesis that H_2_S would protect against the hypoxia-induced defect in proteostasis. For these experiments, we grew YFP::polyQ_35_ animals in 50 ppm H_2_S before exposure to hypoxia (schematized in Fig.6A). This concentration of H_2_S activates HIF-1 and extends lifespan in *C. elegans* (Miller & Roth, 2007; Budde & Roth, 2010). We observed significantly fewer YFP::polyQ_35_ foci in animals exposed to 1000 ppm O_2_ that were grown in H_2_S (Fig.6B). The improvement in proteostasis is functionally important, as we also measured a significant delay in paralysis after exposure to 1000 ppm O_2_ in YFP::polyQ_40_ animals raised in H_2_S relative to untreated controls (Fig.6C). We conclude that pretreatment with H_2_S enhances the ability to maintain proteostasis when challenged with hypoxia. More generally, these data indicate that, as in mammals, adaptation to H_2_S can protect against the effects of hypoxia in *C. elegans*.

One curious aspect of our results is the effect of H_2_S to increase lifespan appears to be distinct from its modulation of proteostasis in hypoxia. We noticed continuous exposure to H_2_S is not required for the effects on proteostasis, although it is for increased lifespan [Fig.6B,C (Miller & Roth, 2007)]. We also observed that treatment with H_2_S for 48 h starting at adulthood is insufficient to protect against age-induced paralysis in YFP::polyQ_40_ animals (Fig.6D). Moreover, SIR-2.1, the *C. elegans* homolog of the sirtuin SIRT1 that is required for the effects of H_2_S on lifespan and thermotolerance (Miller & Roth, 2007), is not required for H_2_S to protect proteostasis in hypoxia (Fig.6E). Just as in wild-type animals, *sir–2.1(ok434)* mutant animals grown in H_2_S develop significantly fewer YFP::polyQ_35_ foci in hypoxia than controls grown in the absence of H_2_S. Taken together, these results suggest that the effects of H_2_S on proteostasis and lifespan are genetically distinct.

**Figure 7:**
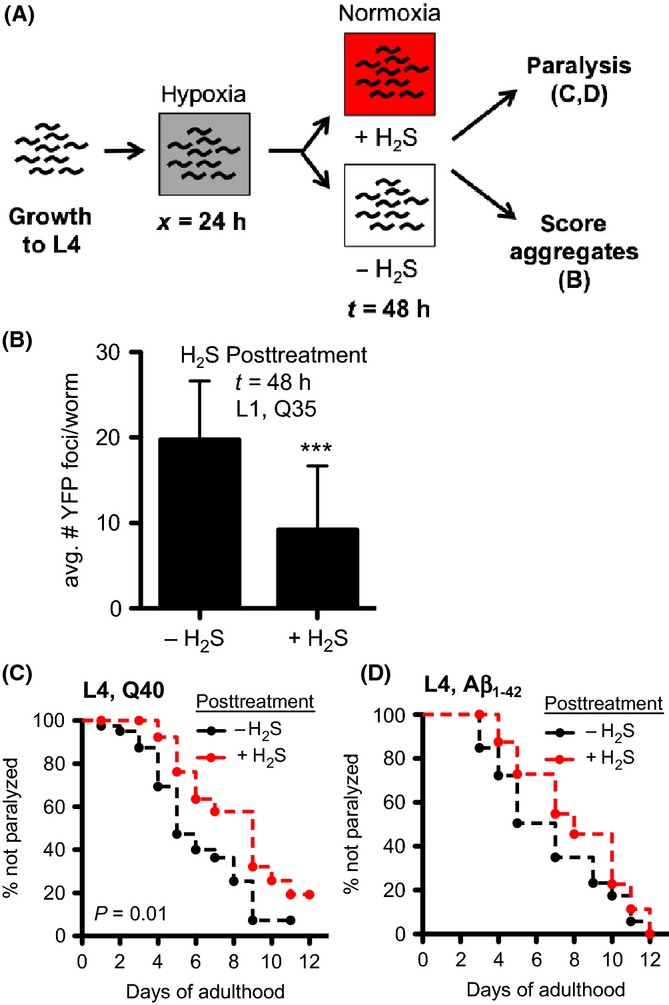
Posttreatment with H_2_S reverses effects of hypoxia on YFP::polyQ_x_ aggregation and toxicity. (A) YFP::polyQ_x_ animals were grown in normoxia (without H_2_S) and then exposed to 1000 ppm O_2_ (*x *=* *24 h). The animals were returned to normoxia to recover ± 50 ppm H_2_S (*t* = 48 h). (B) Recovery in H_2_S slows polyglutamine protein aggregation after return to normoxia. Statistical comparison between H_2_S-treated and untreated controls: ***P < 0.0005. (C,D) H_2_S posttreatment delays hypoxia-induced proteotoxicity. Paralysis is delayed by exposure to H_2_S after removal from hypoxia in both YFP::polyQ_40_ (C) and Aβ_1–42_ (D) animals. For all panels, each cohort consists of 30–50 animals. Summary of data from replicate experiments is included in Table S7 (Supporting information).

H_2_S has been shown to improve outcome in mammalian preclinical models of severe blood loss and myocardial infarct even when administered after the ischemic event (Predmore & Lefer, 2011; Luan *et al*., 2012). This led us to consider the possibility that H_2_S treatment would be sufficient to reverse this effect of hypoxia on proteostasis. To test this, we grew YFP::polyQ_35_ animals in room air (normoxia, without H_2_S), exposed them to 1000 ppm O_2_, and then allowed to recover in the presence or absence of 50 ppm H_2_S (schematized in Fig.7A). Remarkably, animals treated with H_2_S after exposure to hypoxia developed significantly fewer YFP::polyQ_35_ foci during the recovery period than controls that were not exposed to H_2_S (Fig.7B). Posttreatment with H_2_S also delayed the onset of hypoxia-induced paralysis in both YFP::polyQ_40_ and Aβ_1–42_ animals (Fig.7C,D). These data further support our assertions that hypoxia responses induce defects in proteostasis that persist after the hypoxic insult, but also imply that the detrimental effects of hypoxia on proteostasis are reversible.

## Discussion

Disruption of proteostasis contributes to pathologies associated with aging, neurodegenerative diseases, and cancer. There are well-known responses to hypoxia that might be expected to improve proteostasis, such as reduced global translation and induction of protein chaperones. However, our data reveal that *in vivo* the response to specific hypoxic conditions actively disrupts the integrated proteostasis network. Our results are consistent with observations in mammalian systems that ubiquitinated proteins and transgenically expressed proteotoxic proteins aggregate in neurons after ischemia/reperfusion injury *in vivo* (Hu *et al*., 2000; Unal-Cevik *et al*., 2011). Clinically, stroke is often associated with neurodegenerative sequelae and many studies have found an association between stroke and increased risk of Alzheimer's disease (reviewed in Kelleher & Soiza, 2013).

Our studies provide a unique and powerful model to begin to understand how metabolic and physiological adjustments to hypoxia could have long-lasting cellular consequences with important medical implications. For example, in mouse models of Alzheimer's disease, tau protein continues to aggregate even three months after ischemic injury (Koike *et al*., 2011). Similarly, acute ischemia/reperfusion injury in rat models of kidney transplants results in increased fibrosis and kidney dysfunction that are consistent with long-term physiological and cellular changes (Gueler *et al*., 2004). We showed that it is possible to prevent the hypoxia-induced disruption of proteostasis with H_2_S. H_2_S activates HIF-1 by inhibiting the EGL-9 prolyl hydroxylase (Budde & Roth, 2010; Ma *et al*., 2012). However, it is unlikely that H_2_S acts solely through *hif-1* to improve proteostasis, especially as constitutive activation of HIF-1 does not improve proteostasis in hypoxia. H_2_S exposure also results in the transcriptional upregulation of F-box proteins, which are adaptors for SCF ubiquitin ligases. Thus, it may be that H_2_S protects the proteostasis network against hypoxia-induced protein aggregation by modulating the ubiquitin proteasome system. Nevertheless, our results show that the beneficial effects of H_2_S are conserved from mammals to nematodes, which suggests a fundamental integration of H_2_S signaling and cellular responses to hypoxia.

Our results indicate that hypoxia-induced disruptions of proteostasis are reversible, as treatment with H_2_S after the hypoxic insult is sufficient to reduce both protein aggregation and cytotoxicity. In mammals, postconditioning with H_2_S protects against ischemic injury and severe blood loss (King & Lefer, 2011). Proteostasis decreases with age and contributes to a variety of devastating neurodegenerative diseases. It has been proposed that proteostasis failure is a key driver of the aging process (Morley *et al*., 2002; Douglas & Dillin, 2010; Taylor & Dillin, 2011; O'Neill *et al*., 2012). However, we found that protective effects of H_2_S are independent of *sir-2.1*, which is required for increased lifespan in H_2_S (Miller & Roth, 2007). Moreover, short treatments with H_2_S that do not increase lifespan are sufficient to protect against hypoxia-induced proteostasis. While the maintenance of proteostasis and lifespan has been shown to be tightly linked, our work adds to a growing collection of evidence that these two processes can be decoupled (Christie *et al*., 2014; El-Ami *et al*., 2014). We propose that proteostasis and aging are decoupled by H_2_S and suggest the exciting possibility that other defects in proteostasis may be reversible even in aged organisms.

## Materials and methods

### *C. elegans* strains and methods

Animals were maintained on nematode growth media (NGM) with OP50 *E. coli* at 20°C (Brenner, 1974). For worm strains, see Table S7 (Supporting information).

### Constructing hypoxic and H_2_S-containing environments

Hypoxic and H_2_S conditions were maintained using continuous flow chambers, as previously described (Padilla *et al*., 2002; Fawcett *et al*., 2012). Compressed gas tanks were purchased from Airgas (Seattle, WA) and were certified standard to within 2% of the indicated O_2_ concentration (balanced with N_2_). Hypoxic chambers were maintained in a 20°C incubator for the duration of the experiments. H_2_S was diluted to 50 ppm with house air from a 5000 ppm stock tank (balance N_2_) as previously described (Fawcett *et al*., 2012). H_2_S environments were maintained in a fume hood at room temperature, with matched house-air (without H_2_S) environments. Cultures were maintained continuously in H_2_S for pretreatment experiments.

### YFP::polyQ_x_ aggregation assay

Synchronized cohorts of 50–75 YFP::polyQ_x_ animals were exposed to hypoxic environments for approximately 24 h at 20°C on NGM plates seeded with live OP50 food. Palmitic acid (10 mg mL^−1^ in ethanol) was used to form a physical barrier around the edge of each plate to encourage the animals to remain on the surface of the plate when in hypoxia. To visualize the localization of the YFP, worms were mounted on an agar pad in a drop of 20 mm sodium azide as anesthetic. Control experiments showed that azide did not affect the aggregation of YFP::polyQ_35_ or YFP::polyQ_40_, as observed by Moronetti Mazzeo *et al*. (2012). To evaluate protein aggregation in hypoxia, YFP foci were counted immediately after the hypoxic exposure. YFP foci were identified and quantified as described in Morley *et al*. (2002) and Silva *et al*. (2011). Aggregates were visualized and counted using a Nikon 90i fluorescence microscope with the GFP filter and oil-immersion 20× objective (Nikon Instruments Inc., Melville, NY, USA).

Synchronous YFP::polyQ_40_ populations were generated by allowing first-day adult animals lay eggs for 1 h, after which time the adults were removed and the plates were incubated at 20°C overnight. Cohorts of 50–75 larvae were suspended in M9 and mouth-pipetted to NGM plates for hypoxic exposure. L4 animals were picked from well-fed, logarithmically growing populations and either exposed to hypoxia or allowed to develop to young adults overnight at 20°C.

In all experiments, the number of aggregates was counted blind to treatment. Statistical significance was evaluated by calculating *P*-values using Mann–Whitney nonparametric tests in GraphPad Prism version 5.0d for Mac OS X, GraphPad Software, San Diego California USA, http://www.graphpad.com. In experiments containing more than 2 experimental conditions or strains, a Kruskal–Wallis test and Dunn's multiple comparisons post hoc analysis were performed to calculate the *P*-values between conditions. In experiments with time courses, a two-way paired ANOVA was performed to calculate the *P*-value between time points. In all cases, *P* < 0.05 was considered to be statistically significant. Summary data from replicate experiments are included in Tables S1–S6 (Supporting information).

### Paralysis assays of proteotoxicity

Animals expressing Aβ_1–42_, tau(V337M), or YFP::polyQ_x_ were exposed to 1000 ppm O_2_ for 24 h at 20°C either as L4 or L1. After hypoxic exposure, animals were returned to normoxia and incubated at 20°C. Paralysis was scored daily. Worms were considered paralyzed if they exhibited movement of the nose or tail or pharynx pumping, but remained immobile after tapping with a platinum wire pick 3 consecutive times. Animals that did not move or pump were scored as dead. Dead and bagged worms were censored from the experiment on the day of death/bagging. Paralyzed worms were removed from the plate on the day of paralysis. Live worms that were not paralyzed were moved to a new plate each day until all worms were either scored as paralyzed or dead. Kaplan–Meier log-rank (Mantel–Cox) tests using GraphPad Prism were used to evaluate statistical significance.

Uncoordination in *dyn-1(ky51)* ts mutants was measured as described in Gidalevitz *et al*. (2006). Experiments shown were performed using unseeded NGM plates.

## Supporting Information

**Fig. S1**5000 ppm O_2_ does not accelerate paralysis associated with expression of polyglutamine proteins. **Fig. S2** Exposure to hypoxia does not result in upregulation of genes involved in maintenance of proteostasis. **Fig. S3** Mutation of *hif-1* in YFP::polyQ35 animals does not exacerbate hypoxiainduced proteotoxicity.

**Table S1**Summary of data from replicate experiments as in Fig. 1.

**Table S2**Summary of data from replicate experiments as in Fig. 2.

**Table S3**Summary of data from replicate experiments as in Fig. 3.

**Table S4**Summary of data from replicate experiments as in Fig. 4.

**Table S5**Summary of data from replicate experiments as in Fig. 5.

**Table S6**Summary of data from replicate experiments as in Figs 4 and 6.

**Table S7**Summary of data from replicate experiments as in Fig. 7.

**Table S8**List of worm strains.

**Table S9**Summary of data from replicate experiments as in Figs 1,2 and 3 (Supporting information).
