# Supplementary material for: Hypoxia disrupts proteostasis in Caenorhabditis elegans
Source: Aging Cell. 2014 Dec 16;14(1):92–101. doi: 10.1111/acel.12301 (PMC4326909; doi:10.1111/acel.12301)
Supplement: Supplementary file 1 [file acel0014-0092-sd1.pdf]

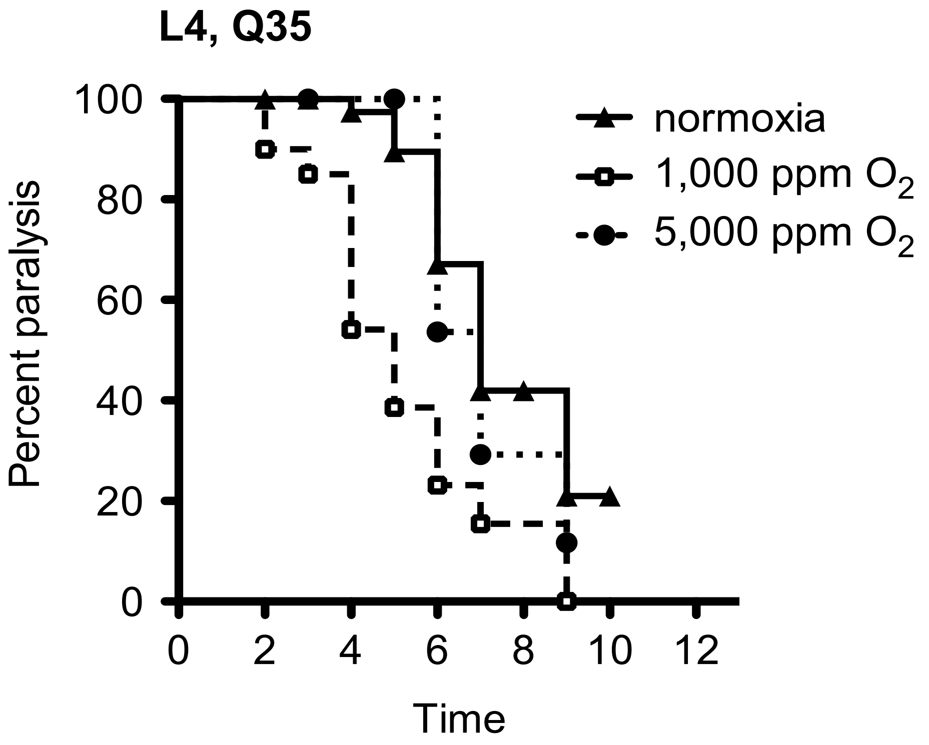

Supplemental Figure 1. 5000 ppm O_2 does not accelerate paralysis associated with expression of polyglutamine proteins. YFP::polyQ_x animals exposed to 5000 ppm O_2 become paralyzed at the same rate as normoxic controls. L4 animals were exposed to 1,000 or 5,000 ppm O_2 for 24 h and then returned to normoxia. Paralysis was scored daily. Each cohort included 30-40 animals. Summary of data from replicate experiments is included in Table S9.

Fawcett et al., Supplemental Figure 2

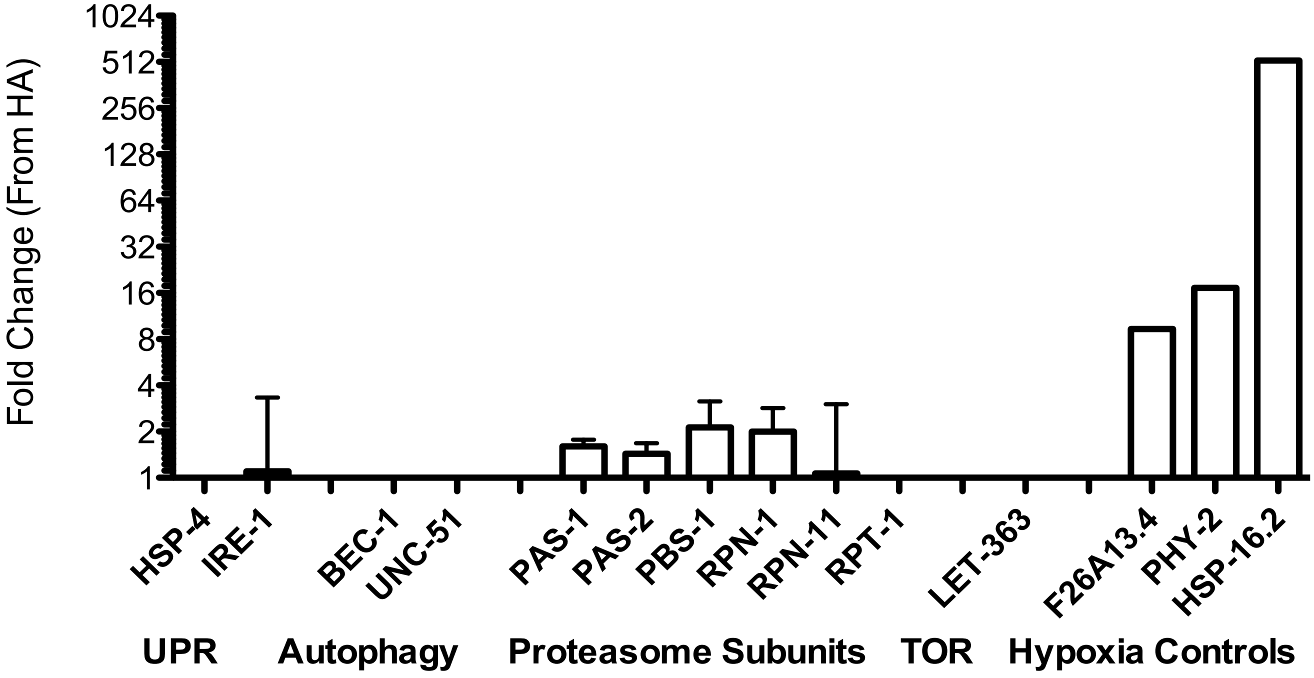

Supplemental Figure 2. Exposure to hypoxia does not result in upregulation of genes involved in maintenance of proteostasis. qRT-PCR analysis of genes commonly identified as upregulated in response to hypoxia. $\Delta\Delta C_t$ were calculated as described in (Miller et al., 2011). Upregulated hypoxia controls were selected from microarray data published in (Shen et al., 2005). 9,000 synchronized L4 animals were exposed to hypoxia for 24 hours and harvested into Trizol. qPCR primers are available upon request.

Fawcett et al., Supplemental Figure 3

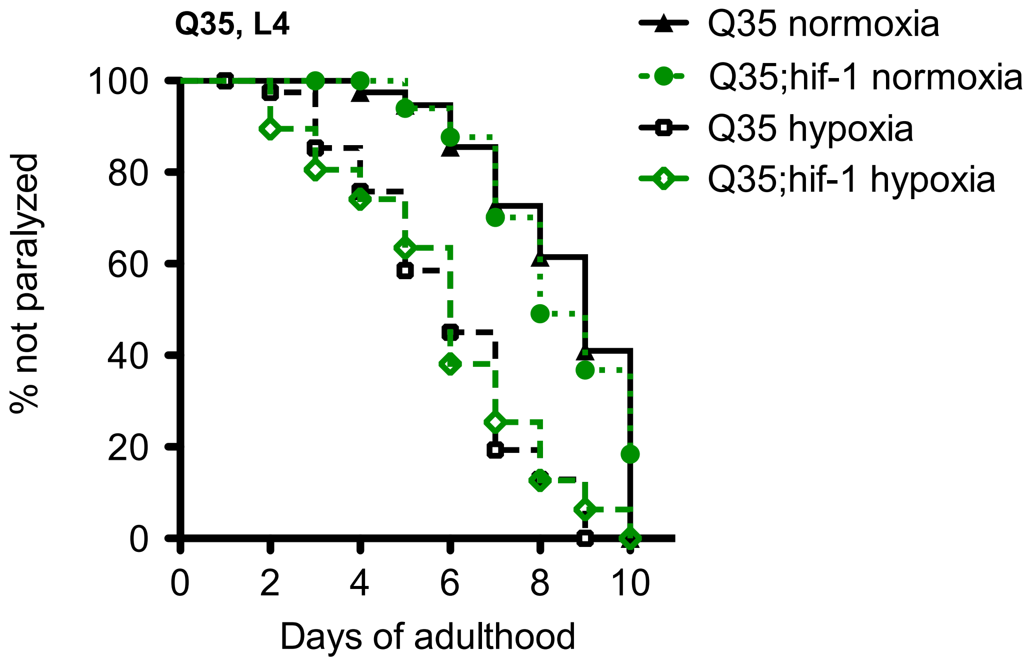

Supplemental Figure 3. Mutation of *hif-1* in YFP::polyQ$_{35}$ animals does not exacerbate hypoxia-induced proteotoxicity. *Hif-1* mutant animals exposed to 1000 ppm O_2 become paralyzed at the same rate as wild type controls. L4 animals were exposed to 1,000 ppm O_2 for 24 h and then returned to normoxia. Paralysis was scored daily. Each cohort included 30-40 animals. Summary of data from replicate experiments is included in Table S9.
