# Supplementary material for: Hypoxia disrupts proteostasis in Caenorhabditis elegans
Source: Aging Cell. 2014 Dec 16;14(1):92–101. doi: 10.1111/acel.12301 (PMC4326909; doi:10.1111/acel.12301)
Supplement: Supplementary file 9 [file acel0014-0092-sd9.docx]

**Table S8: List of worm strains**

| Strain | Reference |
| --- | --- |
| *hif-1(ia04)* | Jiang et al. 2001 |
| *vhl-1*(*ok161*) | Epstein et al. 2001 |
| *egl-9(sa307)* | Epstein et al. 2001 |
| *sir‑2.1*(*ok434*) | Tissenbaum & Guarente 2001 |
| *daf-16(mu89)* | Lin et al. 1997 |
| AM140 *rmls132[p_unc-54_::q35::yfp]* | Satyal et al. 2000 |
| AM141 *rmls133[p_unc-54_::q40::yfp]* | Satyal et al. 2000 |
| *hif-1(ia04); YFP::polyQ_35_* | * |
| *vhl-1(ok161); YFP::polyQ_35_* | * |
| *egl-9(sa307); YFP::polyQ_35_* | * |
| *daf-16(mu86); YFP::polyQ_35_* | * |
| *sir-2.1(ok434); YFP::polyQ_35_* | * |
| CL2006 *dvls2[P_unc-54_::beta-peptide;pRF4]* | Link, 1995 |
| CK10 *bkIs10[P_aex-3_::h4R1NTauV337M;P_myo-2_::gfp]* | Kraemer et al., 2003 |
| *dyn-1(ky51)* | Clark et al. 1997 |

- Strains were generated by crossing AM140 or AM141 with each genetic background using standard techniques (Brenner 1974). Mutant alleles were followed by reported phenotype or PCR genotyping. Primer sequences are available upon request.
